# Exploring the longitudinal associations between census division income inequality and BMI trajectories among Canadian adolescent: Is gender an effect modifier?

**DOI:** 10.1016/j.ssmph.2023.101519

**Published:** 2023-09-21

**Authors:** Samuel A.J. Lowe, Stephen Hunter, Karen A. Patte, Scott T. Leatherdale, Roman Pabayo

**Affiliations:** a School of Public Health, University of Alberta, Edmonton, AB, Canada; b Faculty of Applied Health Sciences, Brock University, St. Catharines, ON, Canada; c School of Public Health Sciences, University of Waterloo, Waterloo, ON, Canada

**Keywords:** Income inequality, Adolescent health, Social epidemiology, Obesity, Overweight, BMI

## Abstract

**Background:**

Income inequality is a structural determinant of health linked to increased risk of overweight and obesity, although its links to the health of adolescent populations are not well understood. This study investigated the longitudinal associations between census-division-level (CD) income inequality and BMI trajectories among Canadian adolescents, and determine if these associations vary by gender.

**Methods:**

Study data are from the Cannabis use, Obesity, Mental health, Physical Activity, Alcohol use, Smoking, and Sedentary behaviour (COMPASS) cohort of adolescents attending secondary schools in Canada. Our sample included 14,675 adolescents who were followed up across three waves of the COMPASS study (2016–2017, 2017–2018, and 2018–2019) and linked to 30 CDs. Measures of income inequality and other area-level covariates were derived and linked to COMPASS participants using data from the 2016 Canadian Census. We utilized multilevel mixed-effects linear regression modelling to quantify the associations between income inequality and BMI and test for effect modification by gender. Sensitivity analyses were run excluding those with BMI scores in the range considered overweight or obesity at baseline.

**Results:**

Higher CD income inequality was significantly associated with higher z-transformed BMI scores (β = 0.11, 95% CI = 0.034 to 0.19). The interaction term between income inequality and time was not statistically significant, indicating that this association remained constant over time. Once stratified by gender, the association between inequality and BMI became stronger for males (β = 0.14, 95% CI = 0.060 to 0.022) and attenuated for females (β = 0.063, 95% CI = −0.047 to 0.17).

**Conclusion:**

Attending schools in CDs with higher income inequality was associated with higher BMI scores among male but not female adolescents. Further work is needed to investigate this discrepancy and identify the structural mechanisms that mediate the relationship between inequality and adolescent health.

## Highlights

• Income inequality has been linked to an increased risk of overweight and obesity.
• This relationship has not been well-studied among adolescents over time.
• Higher census division income inequality was associated with higher BMI scores.
• When stratified by gender, this relationship persisted for males but not females.
• Future studies should clarify the mechanisms linking income inequality to health.

## 1 Introduction

The increasing global burden of overweight and obesity, especially among youth and adolescents, highlight an ongoing challenge central to public health (Bancej et al., 2015; Fan & Zhang, 2022; Lobstein, Baur, & Uauy, 2004; Murphy et al., 2018; Obesity and Overweight, 2021; Rao, Kropac, Do, Roberts, & Jayaraman, 2016). The World Health Organization (WHO) estimates that as many as 340 million youth and adolescents worldwide, aged 5–19 were at risk of overweight or obesity in 2016 (Obesity and Overweight, 2021). A recent meta-analysis of 21 countries reporting a pooled prevalence of overweight and obesity of 24.4%–27.7% among adolescents aged 12 to 15 (Fan & Zhang, 2022). In Canada, the prevalence of obesity has doubled among Canadian youth and adolescents from 6.3% in 1978/79 to 13.3% in 2004 for ages 2 to 17, and this burden is projected to remain as high as 14.3% until 2031 (Bancej et al., 2015; Rao et al., 2016). Overweight and obesity is conceptualized by the WHO as abnormal or excessive fat accumulation, and often measured using the Body Mass Index (BMI) using a threshold of one standard deviation (overweight) and two standard deviations (obese) above the WHO Growth Reference median (Obesity and Overweight, 2021). High BMI has been identified as a major risk factor for noncommunicable diseases and mortality. For example, overweight and obesity in adolescence increases the risk of type II diabetes, hypertension, depression, and lower educational attainment both in the short term and into adulthood (Herman, Craig, Gauvin, & Katzmarzyk, 2009; Lobstein et al., 2004; Obesity and Overweight, 2021; Rao et al., 2020).

Despite widespread consensus that the drivers of weight-related health disparities are complex and multifactorial (Nobles, Summerbell, Brown, Jago, & Moore, 2021), a large proportion of existing research, policy, and interventions focus on individual behaviours and predictors (Ramos Salas, Forhan, Caulfield, Sharma, & Raine, 2017; Schuler, Vazquez, & O’Reilly, 2023). A Cochrane Review of 153 randomized control trials assessing interventions for preventing obesity in children found that the majority of interventions (n = 140, 57.9%) targeted individual lifestyle factors, and that this individual-level focus has persisted relatively unchanged despite the limited effectiveness of these interventions alone in preventing childhood obesity.^9^However, emerging evidence suggests that the broader structural contexts and conditions in which we are born, live, and grow also play a large role in shaping population and individual health (Berkman et al., 2014; Raphael, Bryant, Mikkonen, & Rephael, 2020). Thus, there is a need to supplement our current understanding and efforts in addressing weight-related health disparities by considering upstream structural and systemic determinants.

According to the Social Determinants of Health (SDOH) framework, income inequality – the inequitable distribution of income within a given area – is a structural characteristic of shared environments that shapes the health of youth, adolescents, and adults in a variety of ways (Berkman et al., 2014; Raphael et al., 2020). Income inequality both between and within countries has been steadily increasing over the past decades (Frenette, Green, & Milligan, 2007; Keeley, 2015), with inequality estimates increasing by an average of 5.9% across Canada between 1990 and 2018 (Statistics Canadaa). Researchers estimate that this inequality is driven largely by the concentration of wealth among those at the upper end of the income spectrum, with top 20% of Canadians estimated to possess over 67% of the country's total net worth (Frenette et al., 2007).

Income inequality at various geographic scales is suspected to engender adverse health behaviours and outcomes, such as excess changes in weight, through three broad pathways. First, the social anxiety pathway posits that increasing inequality promotes stressful social comparisons between ‘haves’ and ‘have-nots’ leading to mistrust, stress, shame, and poor health (Berkman et al., 2014; Kawachi & Kennedy, 1999; Layte, 2012). These stressful comparisons present a more proximal pathway that could engender increases in body mass among adolescent through elevated cortisol levels (De et al., 2009a), as well as generate stress around body image and socially-defined ideals (e.g., ‘thinness’) (Chang & Christakis, 2005; Clément, Levasseur, Seetahul, & Piaser, 2021).Second, the social capital pathway asserts that income inequality could erode social cohesion and capital, the level of connectedness and social resources (e.g., emotional and tangible supports) within an area, similarly resulting in stress and adverse outcomes (Berkman et al., 2014; Layte, 2012; Stansfeld, 2009). Clément et al. (2021) proposed that a lack of social capital could lead to a decrease in community physical activity and social gatherings, and alter the access to and consumption of unhealthy foods and alcohol consumption, which have been linked to increasing body mass. Third, the neo-materialist pathway suggests that increasing income inequality in a given area could result in macro-level material deprivation (e.g., food insecurity, less economic opportunities), limited social mobility, and underinvestment in social infrastructure, such as community greenspaces, social and civic services, access to nutrient-rich and healthy foods, which have been show to promote and support health (Berkman et al., 2014; Kawachi & Kennedy, 1999; Layte, 2012). These proposed mechanisms could be especially relevant to overweight and obesity among adolescents, as multiple studies of have linked social anxiety, social cohesion, access (or lack thereof) to recreational facilities, and school food environments to obesity and related risk factors (e.g., disordered eating, alcohol consumption, physical inactivity) among adolescents (Asthana, 2012; de Assis et al., 2022; Spettigue et al., 2020; Utley, Affuso, & Rucks, 2016).

Currently, there is a small body of work exploring the direct associations between income inequality (primarily at the national level) and overweight and obesity among adolescents. A preliminary cross-sectional study of adolescents from the COMPASS study found a curvilinear association between census-division income inequality within Canadian provinces and higher BMI scores among adolescents (Hunter et al., 2023). Conversely, Quon and McGrath (2015) did not observe a statistically significant cross-sectional association between higher province-level income inequality and BMI among Canadian adolescents aged 12 to 17 (Quon & McGrath, 2015). While sparse, some longitudinal ecological evidence has also been generated linking higher levels of between-country (Utley et al., 2016) and within country (Spettigue et al., 2020) with higher weight status. Further research is needed to address this dearth of information, especially at more proximal geographic scales across time at which specific mechanisms of income inequality (i.e., proximal social comparisons and social cohesion) might be more impactful on adolescent health (Berkman et al., 2014; De and Maio, 2014; Layte, 2012).

Additionally, previous work has indicated that the associations between income inequality and weight may vary by gender (Clément et al., 2021; Hunter et al., 2023; Pickett & Wilkinson, 2015). Among COMPASS students, higher income inequality was significantly associated with higher BMI among males (β = 0.07, 95%CI = 0.04, 0.11) but not females (β = 0.02, 95%CI = −0.01, 0.05) in gender-stratified models, with more pronounced negative slopes according to quadratic inequality terms for females (β = −0.05, 95%CI = −0.04, −0.01) compared to makes (β = −0.02, 95%CI = −0.04, −0.01) (Hunter et al., 2023). Based on the proposed pathways linking income inequality and health, heterogeneity between gender could be due to a variety of factors, including differing forms of social networks, references of social comparison, engagement with social infrastructure, and proclivities for harmful coping behaviours (Berk, 2014; Blumenthal, Ham, Cloutier, Bacon, & Douglas, 2016; Colarossi, 2001; De et al., 2009b; Pabayo et al., 2014, 2021; Pompili & Laghi, 2019; Spettigue et al., 2020) However, other cross-sectional evidence is conflicting, with some findings significant associations for both females and males (Due et al., 2009), neither females or males (Elia et al., 2020), or for females but not for males (Murphy et al., 2018). Further longitudinal investigation is needed to clarify if and how the link between income inequality and weight varies by gender over time, especially considering the disparities in obesity burden and differences in gendered social norms and life experiences that males and females experience (Anekwe et al., 2020; Bancej et al., 2015; Kanter & Caballero, 2012; Rao et al., 2016).

Our study sought to build on previous work (Hunter et al., 2023) and address gaps in current knowledge through a longitudinal, multilevel approach to quantify the association between area-level income inequality and BMI among adolescents. Specifically, we assessed whether income inequality at the census-division level was associated with changes in BMI trajectories for students attending secondary school in Canada, and whether this association was heterogeneous across genders.

## 2 Methods

### 2.1 Study sample

Outcome and individual covariate data are from the Cannabis use, Obesity, Mental health, Physical activity, Alcohol use, Smoking, and Sedentary behaviour (COMPASS) study, an ongoing prospective cohort of adolescents attending school in the Canadian provinces of Alberta, British Columbia, Ontario, and Québec (Leatherdale et al., 2014). Students in this prospective cohort (2012–2027) are in grades 9 through 12, or Secondary I through V in Québec. Data on a variety of sociodemographic, physical and mental health, and substance use factors are collected annually via paper based (2012 to March 2020) and online (April 2020 onward) questionnaires. Students were eligible to participate if they attended a participating school that permitted active-information passive parental permission consent protocols.

Due to its longitudinal and quasi-experimental approach, the COMPASS cohort provides an apt data source for examining the associations between contextual social determinants and health over time (Leatherdale, 2018). The current study utilized data from three waves of the COMPASS study, aligning with the 2016–2017, 2017–2018, and 2018–2019 school years prior to the transition to online and remote learning in response to the COVID-19 pandemic.

To capture income inequality and other area-level covariates, COMPASS responses were linked to data from the 2016 Canadian Census using geocoded information from the participating schools. Area-level data were linked to the census division (CD) of each COMPASS school, which refers to an intermediate aggregation level that lies in-between municipal and territorial levels (Statistics Canada, 2022). Many regional planning, resource allocation, and public service provision decisions are made at the CD level, indicating that CDs are an appropriate level for operationalizing and gauging the links between area-level income inequality and health. As many adolescents from across the socioeconomic spectrum spend a large portion of their waking hours in school environments,^45^capturing levels of inequality within the school context relevant for understanding adolescent health and wellbeing.

A case-complete dataset was generated by including students who could be linked to geocoded data and were not missing baseline outcome, exposure, and covariate data in the 2016–2017 wave. Of the 46,862 COMPASS students captured in this wave, our final analytic sample contained 14,675 adolescents attending 76 schools clustered within 30 CDs.

### 2.2 Measures

#### 2.2.1 Area-level covariates

Area-level income inequality for each CD was measured using Gini coefficients, which range from 0 (complete equality) to 1 (complete inequality). The calculation of Gini coefficients has been described previously (von Hippel, Hunter, & Drownb, 2017). Gini coefficients for each CD were calculated using after-tax income data from the 2016 Canadian census and categorized into ‘high’ and ‘low’ groups using the median as a cut-off. Additionally, CD median outcome ($CAD), population size, proportion of visible minority residents, and proportion of household below the low-income cut-off (LICO) (Statistics Canada, 2015) were included as area-level covariates and z-transformed for comparability.

#### 2.2.2 Individual-level covariates

Baseline covariate data included age, binary gender, race/ethnicity, school type (public/private), alcohol consumption, and province of residence for each participant. Weekly spending money was included as a proxy for socioeconomic status, as individual and family level SES data are not collected through COMPASS surveys. Additionally, physical activity level was measured as whether the respondents met Canadian guidelines of at least 60 min of moderate and/or vigorous physical activity per day. Continuous age was mean-centered, with all other variables retained as categorical according to COMPASS response options. A dummy variable for time was created to represent each survey wave.

Of note, COMPASS students were asked “are you male or female”, which likely captures aspects of both sex (i.e., biology) and gender (i.e., social construct) (Johnson, Greaves, & Repta, 2009). As the current study considers BMI in relation to socioeconomic factors and structures, we present all data and findings using the term “gender”. This approach is consistent with prior work on income inequality and health (Horino, Liu, Lee, Kawachi, & Pabayo, 2020; Hunter et al., 2023).

#### 2.2.3 Outcome measures

Body mass index (BMI) for each study participant was calculated by dividing students’ self-reported weight in kilograms (kg) by the square of their height in meters (m^2^). Height and weight data collection items align with those used in the Youth Risk Behaviour Survey, with a validation study using COMPASS data reporting high test-retest reliability (ICC = 0.95) and concurrent validity (ICC = 0.84) for BMI (Leatherdale & Laxer, 2013). We z-transformed BMI scores using a macro STATA package from the WHO that calculates BMI-for-age scores for females and males (de Onis et al., 2007).

#### 2.2.4 Statistical analyses

Multilevel mixed-effects linear regression modelling was used to quantify the associations between area-level income inequality and continuous WHO BMI z-score trajectories using a step-up approach. Intercept-only models were constructed first to calculate the intraclass correlation coefficient (ICC) for BMI, which is a measure of the amount of variability in the outcome that can be attributed to the area and individual levels. These models were then adjusted for CD income inequality and follow-up time to generate crude associations between inequality and BMI at baseline and over time. Individual and area-level covariates were then added to the models to provided fully-adjusted estimates. Finally, a gender*income inequality interaction term was added to the model to assess baseline difference across gender, and if significant, stratified analyses were repeated for male and female participants to assess potential effect modification by gender at baseline and over time.

Initially, models were constructed with four levels of clustering: repeated measures nested within individuals nested within schools nested within CDs. However, variance in BMI at the school level was negligible after adjusting for CD clustering, and so nesting at the school level was omitted in the final models.

A sensitivity analysis was conducted excluding adolescents who had BMIs in the range considered overweight or obesity at baseline (n = 3566), although these results are highly congruent with findings from the above models and are thus not presented here.

Multilevel growth curve modelling allows for the analysis of clustered, hierarchical data, while accounting for missing outcome data over time (Locascio & Atri, 2011). For this study, time was included as both a fixed and random effect, meaning that the intercepts and trajectories of individual adolescents were allowed to vary. All analyses were conducted using STATA MP (V.17.0) using a threshold of p ≤ 0.05 to indicate statistical significance.

### 2.3 Sensitivity analysis

Multilevel models were repeated excluding adolescents who had BMIs in the range considered overweight or obesity at baseline (n = 3566). This sensitivity analysis was conducted to account for the potential influence of elevated baseline BMI scores as a predictor for subsequent elevations in BMI.

## 3 Results

Individual and area-level baseline characteristics of the sample of 14,662 adolescents nested within 30 CDs are presented in Table 1. The majority of adolescents in the sample were white (75.4%), and attending public school (94.5%) in Ontario (71.6%). The sample was relatively balanced in terms of gender (51.9% female) and physical activity (47.2% ≥ 60 min per day). The mean age at baseline was 15.0 years (SD = 1.0 years). Characteristics of the 30 included census divisions are also presented in Table 1. Mean after-tax income inequality was 0.37 (SD = 0.022, range = 0.30–0.46), which is relatively high compared to 2016 estimates for Alberta (0.296), British Columbia (0.298), Ontario (0.318), and Québec (0.283) (Statistics Canadab).

**Table 1:** Baseline (2016–2017) sociodemographic and census division characteristics of adolescents from the COMPASS study (n = 14,662).

Individual Characteristics	All Adolescents	Females	Males
	n (%)	n (%)	n (%)
Gender			
Female	7605 (51.9)	7605 (100.0)	–
Male	7057 (48.1)	–	7057 (100.0)
Weekly Spending Money			
0$	2419 (16.5)	1133 (14.9)	1286 (18.2)
$1-$5	1060 (7.2)	578 (7.6)	482 (6.8)
$6-$10	1295 (8.8)	679 (8.9)	616 (8.7)
$11-$20	2255 (15.4)	1206 (15.9)	1049 (14.9)
$21-$40	1834 (12.5)	955 (12.6)	879 (12.5)
$41-$100	1783 (12.2)	1012 (13.3)	771 (10.9)
>$100	1916 (13.1)	853 (11.2)	1063 (15.1)
Do Not Know	2100 (14.3)	1189 (15.6)	911 (12.9)
Race/Ethnicity			
White	11,059 (75.4)	5739 (75.5)	5320 (75.4)
Black	410 (2.8)	187 (2.5)	223 (3.2)
Asian	886 (6.0)	478 (6.3)	408 (5.8)
Latin American	299 (2.0)	135 (1.8)	164 (2.3)
Other	808 (5.6)	398 (5.2)	410 (5.8)
Multiracial	1200 (8.2)	668 (8.8)	532 (7.5)
Province			
Alberta	855 (5.8)	433 (5.7)	422 (6.0)
British Columbia	748 (5.2)	385 (5.1)	363 (5.1)
Ontario	10,502 (71.6)	5319 (69.9)	5183 (73.4)
Québec	2557 (17.4)	1468 (19.3)	1089 (15.4)
School Type			
Public	13,861 (94.5)	7191 (94.6)	6670 (94.5)
Private	801 (5.5)	414 (5.4)	387 (5.5)
Alcohol Consumption			
< Once per Month	10,561 (72.0)	5611 (73.8)	4950 (70.1)
Monthly	3169 (21.6)	1617 (21.3)	1552 (22.0)
Weekly	932 (6.4)	377 (4.9)	555 (7.9)
Physical Activity			
<60 Minutes per Day	7748 (52.8)	4714 (62.0)	3034 (43.0)
≥60 Minutes per Day	6914 (47.2)	2891 (38.0)	4023 (57.0)
**Mean (SD; Range)**			
Age (years)	15.0 (1.0; 13–18)	15.0 (1.0; 13–18)	15.0 (1.0; 13–18)
**Census Division Characteristics (n=30)**	**Mean (SD)**	**Range**	
Income Inequality	0.37 (0.022)	0.30–0.46	
LICO (%)	6.6 (2.5)	1.8–13.9	
Median Income ($CAN)	62,888 (8875)	49,203–87,183	
Visible Minority (%)	9.8 (10.1)	0.6–49.2	
Population Size	352,292 (362,803)	15,509–2,463,431	

The average BMI score at baseline was 21.6 (SD = 4.1) and ranged from 11.1 to 49.6. Average BMI scores remained relative constant over time, increasing slightly to 22.2 (SD = 4.0, range = 12.1–49.6) during wave 2 and 22.4 (SD = 4.1, range = 11.7–49.8).

In terms of BMI categories, 2.3% (n = 342) adolescents were classified as underweight, 73.4% (n = 10,756) as ‘healthy weight’, 16.7% (n = 2444) as overweight, and 7.6% (n = 1120) as at risk of obesity. Similarly, 15.9% (n = 1831) and 7.4% (n = 850) adolescents follow-up at wave two had BMI classifications of overweight and obesity, respectively. At wave three, 15.7% (n = 1084) had BMIs classified as overweight and 7.1% (n = 488) as at risk for obesity.

**Fig. 1:**
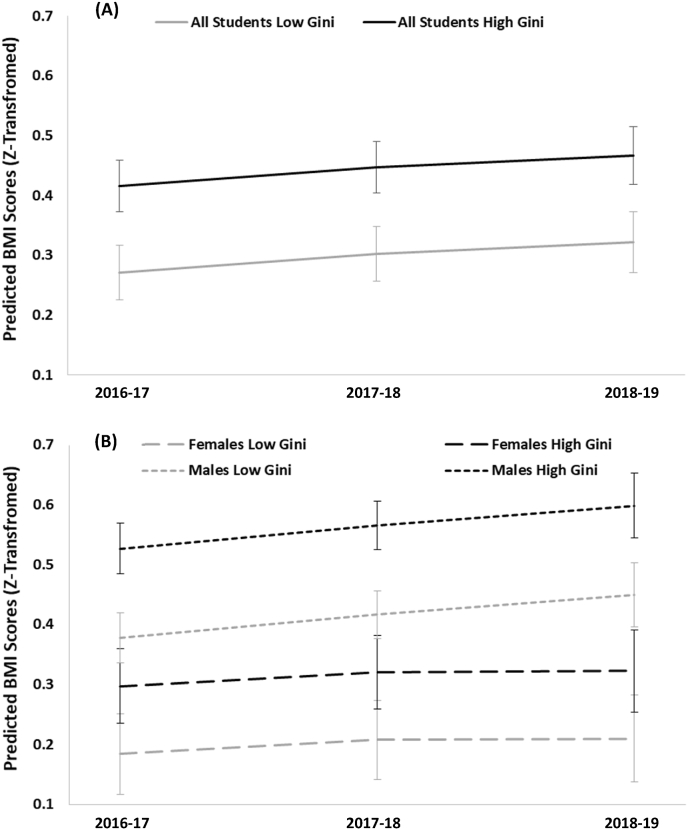
Predicted trajectories of BMI scores among (**A**) all adolescents (n = 14,662) and (**B**) female (n = 7605) and male (n = 7057) adolescents from three waves of the COMPASS study (2016–2019).

The intercept-only model for BMI yielded an ICC value of 0.021, indicating that 2.1% of the variation in BMI could be attributed to the CD level. After adding Gini time, and individual and area-level covariates, the fully-adjusted association between income inequality and BMI was statistically significant (β = 0.11, 95% CI = 0.034 to 0.19). Thus, adolescents attending schools in CDs with high income inequality had higher average BMI scores at baseline and over time compared to those attending schools in CDs with low inequality (Table 2, Fig. 1). The interaction term for Gini and the second wave (β = −0.024, 95% CI = −0.049 to 0.0024) and third wave (β = −0.027, 95% CI = −0.062 to 0.0086) were not statistically significant, indicating that the influence of inequality on BMI remained constant over time (Table 2).

**Table 2:** Fully**-**adjusted associations between individual and neighbourhood characteristics and BMI for all students, as well as stratified by gender, for 14,662 adolescents nested within 30 census divisions from the COMPASS study (2016-17 to 2018-19).

	BMI – Full Sample (n = 14,662) β (95%CI)	BMI – Females (n = 7605) β (95%CI)	BMI – Males (n = 7057) β (95%CI)
**FIXED EFFECTS**			
**Income Inequality and Time**			
Gini (ref: Low Inequality)			
High Inequality	**0.11 (0.034, 0.19)**	0.063 (−0.047, 0.17)	**0.14 (0.060, 0.22)**
Year (ref: 2016–2017)			
2017–2018	**0.042 (0.018, 0.066)**	**0.032 (0.0013, 0.062)**	**0.053 (0.017, 0.090)**
2018–2019	**0.062 (0.023, 0.10)**	0.030 (−0.021, 0.080)	**0.092 (0.032, 0.15)**
Gini*Year			
High Inequality*2017–2018	−0.024 (−0.049, 0.0024)	−0.018 (−0.051, 0.015)	−0.031 (−0.071, 0.0095)
High Inequality*2018–2019	−0.027 (−0.062, 0.0086)	−0.010 (−0.054, 0.034)	−0.046 (−0.10, 0.0098)
**Area-Level Characteristics**			
LICO (%)	−0.050 (−0.11, 0.0080)	−0.033 (−0.12, 0.050)	**−0.065 (-0.12, -0.0061)**
Median Income (%)	−0.062 (−0.12, 0.00069)	−0.057 (−0.14, 0.030)	−0.050 (−0.12, 0.018)
Visible Minority (%)	−0.019 (−0.095, 0.057)	0.023 (−0.086, 0.13)	−0.052 (−0.12, 0.016)
Population Size	0.0080 (−0.053, 0.069)	−0.040 (−0.13, 0.048)	0.036 (−0.024, 0.096)
**Individual-Level Characteristics**			
Age	**−0.034 (-0.051, -0.018)**	−0.0015 (−0.023, 0.020)	**−0.066 (-0.091, -0.041)**
Gender (ref: Female)			
Male	**0.21 (0.17, 0.24)**	–	–
Weekly Spending Money (ref: $0)			
$1-$5	0.0047 (−0.036, 0.045)	0.019 (−0.033, 0.071)	−0.0083 (−0.072, 0.055)
$6-$10	0.029 (−0.0088, 0.066)	0.013 (−0.035, 0.061)	0.045 (−0.013, 0.10)
$11-$20	0.010 (−0.022, 0.042)	0.0098 (−0.032, 0.052)	0.010 (−0.039, 0.059)
$21-$40	0.021 (−0.012, 0.054)	−0.011 (−0.054, 0.032)	**0.057 (0.0054, 0.11)**
$41-$100	**0.037 (0.0052, 0.068)**	0.012 (−0.028, 0.053)	**0.065 (0.015, 0.11)**
>$100	**0.034 (0.0036, 0.065)**	0.025 (−0.017, 0.066)	0.044 (−0.0021, 0.090)
Do Not Know	0.011 (−0.021, 0.043)	0.024 (−0.017, 0.065)	−0.000088 (−0.050, 0.049)
Race/Ethnicity (ref: White)			
Black	**0.14 (0.065, 0.22)**	**0.18 (0.055, 0.31)**	**0.12 (0.021, 0.23)**
Asian	**−0.071 (-0.13, -0.0095)**	**−0.10 (-0.19, -0.020)**	−0.032 (−0.12, 0.060)
Latin Canadian	0.077 (−0.011, 0.17)	0.041 (−0.081, 0.16)	0.10 (−0.025, 0.23)
Other	0.045 (−0.0043, 0.095)	0.052 (−0.016, 0.12)	0.042 (−0.030, 0.11)
Multiracial	**0.048 (0.011, 0.085)**	0.041 (−0.0074, 0.089)	0.057 (−0.00049, 0.11)
Province (ref: Ontario)			
Alberta	0.088 (−0.038, 0.21)	0.069 (−0.11, 0.25)	0.082 (−0.063, 0.23)
British Columbia	−0.11 (−0.24, 0.030)	−0.061 (−0.26, 0.14)	−0.13 (−0.26, 0.0038)
Québec	**−0.31 (-0.41, -0.20)**	**−0.21 (-0.35, -0.063)**	**−0.41 (-0.52, -0.29)**
School Type (ref: Public)			
Private	−0.092 (−0.19, 0.0083)	−0.11 (−0.24, 0.015)	−0.083 (−0.22, 0.057)
Alcohol Consumption (ref: < Once per Month)			
Monthly	**0.056 (0.035, 0.077)**	**0.038 (0.012, 0.065)**	**0.072 (0.040, 0.10)**
Weekly	**0.085 (0.052, 0.12)**	**0.059 (0.013, 0.11)**	**0.11 (0.066, 0.16)**
Physical Activity (ref: <60 Minutes per Day)			
≥60 Minutes per Day	0.000081 (−0.017, 0.018)	−0.0089 (−0.032, 0.014)	0.0078 (−0.019, 0.035)
**RANDOM EFFECTS**			
Time (year)	0.040 (0.035, 0.047)	0.030 (0.023, 0.038)	0.051 (0.042, 0.063)

Note: **Bolded values** indicate statistical significance at p ≤ 0.05.

The interaction term for Gini and gender was statistically significant (β = 0.12, 95% CI = 0.046 to 0.19) and so we conducted stratified analyses by gender (Table 2). Once stratified, the association between CD income inequality and BMI was stronger among males (β = 0.14, 95% CI = 0.060 to 0.022) and became attenuated for females (β = 0.063, 95% CI = −0.047 to 0.17), indicating that only male adolescents experienced significant associations between income inequality and BMI at baseline that persisted over time. The interaction terms for Gini and study wave were not significant for males of females, indicating that the longitudinal trajectories of BMI were not modified by gender.

## 4 Discussion

The current study is one of the first to investigate the longitudinal association between CD-level income inequality and BMI trajectories among a large cohort of female and male Canadian adolescents. Our results provide evidence that attending schools in areas with relatively high levels of income inequality is significantly related to increasing higher BMI scores that persist over time. When stratified by gender, this association was shown to be driven solely by male adolescents, as income inequality was not significantly associated with BMI among females.

Findings are consistent with previous studies that found associations between higher levels of income inequality and higher BMI and overweight/obesity prevalence (Due et al., 2009; Elgar et al., 2015; He, James, Merli, & Zheng, 2014; Hunter et al., 2023; Murphy et al., 2018). In particular, a cross-section study of COMPASS students found that higher CD Gini scores were significantly associated with higher BMI scores among the full sample and male adolescents (Hunter et al., 2023). Similarly, a national-level study found that among higher income countries, higher inequality had a stronger association for males compared to females (Due et al., 2009). Adolescents living in areas of widening income inequality could be experiencing unfavourable social comparisons, stigma and bullying and social exclusion, leading to shame, stress and heightened cortisol levels, isolation, and depression (Benny, Patte, Veugelers, Leatherdale, & Pabayo, 2022; Berkman et al., 2014; De et al., 2009a; Kawachi & Kennedy, 1999; Patel et al., 2018). The gradual erosion of social cohesion and social capital could prevent adolescents from accessing needed social and tangible supports, thus leading to maladaptive coping strategies, such as binge eating and risky alcohol consumption (Blumenthal et al., 2016; Pompili & Laghi, 2019; Spettigue et al., 2020). Additionally, while schools present an ideal environment for implementing health interventions due to their ability to target students directly (Barry, Clarke, & Dowling, 2017; Pabayo, Benny, Veugelers, Senthilselvan, & Leatherdale, 2022) and as students spend a large portion of their time there (OECD, 2020), underinvestment and material deprivation in high inequality areas could limit the schools’ ability to provide adequate resources and supports. Subsequent mediation analyses are needed to assess the role of these proposed mechanisms in shaping the pathways between area-level income inequality and BMI among adolescents.

Contrary to our findings that income inequality is only associated with changes in BMI among males, prior multi-country studies of adolescents reported associations between inequality and body weight for both females and males (Due et al., 2009), neither females or males (Elia et al., 2020), or for females but not for males (Murphy et al., 2018). As these studies all considered cross-sectional measures of national-level income inequality, it is likely that they captured different contextual effects and mechanisms of inequality on weight status (Berkman et al., 2014; De and Maio, 2014; Layte, 2012). While national-level inequality might influence overweight and obesity status across genders, as was found by Due et al. (2009), through structural pathways (e.g., material deprivation, limited social infrastructure), inequality at more proximal scales may exert differential influences on weight status through gendered experiences related to social comparisons and social cohesion. Elia et al. (2009) reported that GDP per capita, but not national Gini index, was associated with weight status (Elia et al., 2020), which could indicate that absolute income is more influential for adolescent weight status than relative inequality in Latin American and Caribbean countries. These results are reinforced by Du et al. (2009), who reported an association between higher inequality and higher prevalence of overweight among high-income countries, but no association in their pooled analysis of all included countries (Due et al., 2009). Both of these studies also considered the prevalence of overweight and obesity as dichotomous outcomes (Due et al., 2009; Elia et al., 2020), which fail to capture potentially differential associations between inequality and more subtle fluctuations in continuous BMI. Further, Murphy et al., (2018) proposed that their observed association for female but not male adolescent could be due to inequality prompting changes in BMI earlier in adolescence for females (Murphy et al., 2018). As our study considered a broader age range of adolescents (13–18 years), it is possible that the influences of inequality do not persist for females and emerge in males as they age into later adolescence.

These discrepancies with previous studies could also be due to variations in gendered norms, experiences, and sociocultural contexts that impact males and females differently. One study of European countries found that males were more likely to compare themselves to those outside of their home environments, although the sample was not limited to adolescents (Pompili & Laghi, 2019). For adolescents specifically, some evidence suggests that female adolescents cultivate social networks centered around connection and closeness that include a higher number of supportive friends, while males establish less explicitly supportive networks with greater focus on social status and accomplishments (Berk, 2014; Colarossi, 2001) Further, male adolescents may be more prone to perceiving negative interactions with their friends.^33^So, as income inequality promotes stressful social comparisons and breaks down social cohesion within and around school environments, male adolescents could be at greater risk of engaging in unfavourable comparisons relating to feelings of inferiority, failure, and lack of social support (Berk, 2014; Hunter et al., 2023). Additionally, evidence suggests that school-aged males engage in higher levels of alcohol consumption compared to females (Anekwe et al., 2020; Kanter & Caballero, 2012; Lowe, Basnet, Leatherdale, Patte, & Pabayo, 2023-a), indicating that they may be more likely to engage in unhealthy coping habits such as binge drinking, which has been linked to higher area-level income inequality (Lowe et al., 2023-a).

The variance of BMI at the CD level in our study was relatively low, indicating that income inequality and other area-level factors only accounted for a small proportion of the variance in BMI. However, as a characteristic of our shared socioeconomic environments, income inequality is a ubiquitous exposure that can have large population-level impacts for those across socioeconomic gradients (Berkman et al., 2014; Kawachi & Kennedy, 1999). Further, recent work with COMPASS participants has linked high CD income inequality with BMI and weight status cross-sectionally, as well as various related and interconnected adverse outcomes including depression and psychosocial wellbeing (Benny et al., 2022) alcohol consumption (Lowe et al., 2023-a), and bullying victimization and perpetration (Pabayo et al., 2022). Finally, the causes of obesity and changes in BMI are complex and multifactorial (Nobles et al., 2021), suggesting that income inequality represents one important structural factor within a broader etiological framework. Thus, when taken together with existing evidence, the current study provides support that efforts to shift the distributions of income in a more equitable (or, ‘healthy’) direction could have substantial health benefits for adolescent populations and help prevent further widening of gendered health inequities (ROSE, 1985).

A key strength of this study is its use of longitudinal data of adolescents, which includes three COMPASS survey waves over four years. By modelling trajectories of BMI scores over time, we were able to establish temporality between exposure to area-level income inequality and changes in BMI, as well as account for varying trajectories in BMI within and between adolescents over time (Locascio & Atri, 2011). Additionally, the length of follow-up allowed us to account for potential lag effects between income inequality and health, with limited evidence suggesting that the impacts of income inequality could peak after five to seven years of previous exposure (Zheng, 2012). By considering income inequality as a structural determinant of health, this paper highlights the importance of considering upstream contextual factors contributing to weight related health outcomes and disparities. Operationalizing income inequality CD level specifically allowed us to consider a geographic scale especially relevant for service provision and structural resource allocation (Benny et al., 2022; Pabayo et al., 2022; Statistics Canada, 2022). Finally, by situating our analyses within the context of the SDOH framework, our results highlight heterogeneous influences across genders, suggesting that male and female adolescents may benefit from different approaches and intervention to mitigate the adverse health impacts of inequality.

The following study limitations should also be considered when interpreting our findings. By operationalizing income inequality using the 2016 census, we were unable to account for potential changes in income inequality over time. However, some evidence suggests that exposure to income inequality from up to 15 years prior remains associated with adverse health (Blakely, 2000). As data on COMPASS participants' residences were not available, we were unable to account for the influence of neighbourhood and community income inequality for those living outside their school's CD. The use of self-reported measures of height and weight to calculate BMI could be a source of information bias in this study, although prior validation work supports the robustness of these measures within the COMPASS cohort (Leatherdale & Laxer, 2013). The use of BMI as a measure of weight also presents intrinsic limitations, including its inability to distinguish between weight from excess fat, muscle, or bone mass, and its variability across demographic factors, such as age, sex, and ethnicity (CDC, 2011). Our inability to account for potential confounders including household income, sedentary behaviours, and social interactions and comparisons via social media, as well as a reliance on weekly spending money as a proxy for socioeconomic status, could have introduced residual confounding by failing to capture the full influence of individual and family SES. School recruitment and retention in the COMPASS study could been depending on factors including resources and competing research demands (Leatherdale et al., 2014) thus introducing a potential selection bias. Finally, as more comprehensive responses for gender were not implemented until the 2019–2020 wave of the COMPASS study, our analyses were unable to fully distinguish between ‘sex’ and ‘gender’ and explore the nuances of gendered experiences beyond the female/male binary.

## 5 Conclusion

The current study provides evidence linking area-level income inequality and BMI among Canadian adolescents over time. This work builds upon the foundation of cross-sectional research linking CD income inequality to adolescent BMI (Hunter et al., 2023), and serves as part of a larger project investigating how the health and social wellbeing of adolescents are being influenced by income inequality across Canada (WCHRI grant #3161). Our results highlight that higher income inequality is linked to higher BMI trajectories among male but not female adolescents, which could be due to differences in gendered norms and social contexts. Future research should explore this heterogeneity in greater depth to better understand and address gender health disparities, both by including more definitive and nuanced gender data and by conducting mediation analyses to investigate the pathways linking income inequality to adolescent health behaviours and outcomes. Additionally, similar studies considering smaller geographic areas such as neighbourhoods could provide more comprehensive insights into how contextual socioeconomic inequalities impact the health trajectories of adolescents. Finally, structural interventions and policies that address the specific experiences and needs of adolescents of all genders should be considered in tandem with proximal interventions to address the contextual drivers of health disparities in a more comprehensive and holistic way.

## Ethics statement

Ethics approval for this study was provided by the University of Alberta's Research Ethics Board (project ID Pro 00102121). All procedures received ethics approval from the University of Waterloo (ORE#30118), Brock University (REB#18-099), CIUSSS de la Capitale-Nationale–Université Laval (#MP-13-2017-1264), and participating school boards, including the use of active-information passive-consent parental permission protocols.

## Author statement

SAJL contributed all aspects of the manuscript, including Conceptualization; Data curation; Formal analysis; Funding acquisition; Investigation; Methodology; Project administration; Resources; Software; Supervision; Validation; Visualization; Roles/Writing - original draft; Writing - review & editing. SH, KAP, and STL contributed Conceptualization; Data curation; Methodology; Project administration; Resources; Validation; Writing - review & editing. KAP and STL also contributed to funding acquisition. RP contributed to Conceptualization; Data curation; Funding acquisition; Investigation; Methodology; Project administration; Resources; Software; Supervision; Validation; Visualization; Writing - review & editing.

## Funding

This work was supported by the Women and Children's Health Research Institute (grant #3161; awarded to RP). The COMPASS study has been supported by a bridge grant from the CIHR Institute of Nutrition, Metabolism and Diabetes (INMD) through the “Obesity – Interventions to Prevent or Treat” priority funding awards (OOP-110788; awarded to STL), an operating grant from the CIHR Institute of Population and Public Health (IPPH) (MOP-114875; awarded to STL), a CIHR project grant (PJT-148562; awarded to STL), a CIHR bridge grant (PJT-149092; awarded to KAP/STL), a CIHR project grant (PJT-159693; awarded to KAP), and by a research funding arrangement with Health Canada (#1617-HQ-000012; contract awarded to STL), a CIHR-Canadian Centre on Substance Use and Addiction (CCSA) team grant (OF7 B1-PCPEGT 410-10-9633; awarded to STL), a project grant from the CIHR Institute of Population and Public Health (IPPH) (PJT-180262; awarded to STL and KAP). A SickKids Foundation New Investigator Grant, in partnership with CIHR Institute of Human Development, Child and Youth Health (IHDCYH) (Grant No. NI21-1193; awarded to KAP) funds a mixed methods study examining the impact of the COVID-19 pandemic on youth mental health, leveraging COMPASS study data. The COMPASS-Quebec project additionally benefits from funding from the Ministère de la Santé et des Services sociaux of the province of Québec, and the Direction régionale de santé publique du CIUSSS de la Capitale-Nationale Dr. Roman Pabayo is a Tier II Canada Research Chair in Social and Health Inequities throughout the Lifespan. Dr. Karen Patte is a Canada Research Chair in Child Health Equity and Inclusion.

## Declaration of competing interest

None of the manuscript authors have any competing interests to declare.

## Data Availability

COMPASS study data is available upon request through completion and approval of an online form: https://uwaterloo.ca/compass-system/information-researchers/data-usage-application. The datasets used during the current study are available from the corresponding author on reasonable request.
